# Characteristics and prognosis of pelvic Ewing sarcoma: a SEER population-based study

**DOI:** 10.7717/peerj.7710

**Published:** 2019-09-17

**Authors:** Li Chen, Cheng Long, Jiaxin Liu, Fei Xing, Xin Duan

**Affiliations:** Department of Orthopedics, West China Hospital, Sichuan University, Chengdu, China

**Keywords:** Pelvic Ewing sarcoma, Nomogram, Prognosis, SEER

## Abstract

**Background:**

The pelvis is one of the primary sites of Ewing sarcoma (ES) and is associated with poorer prognoses than the extremities. Due to the rarity of this disease and limited data available, the prognostic factors of pelvic ES remain controversial. Thus, this study aimed to identify independent prognostic factors, and develop a nomogram for predicting survival rates in patients with pelvic ES.

**Methods:**

Using data provided by the Surveillance, Epidemiology, and End Results (SEER) database, variables including age, sex, race, tumor size, tumor stage, surgery, and radiotherapy were analyzed using the Kaplan–Meier method and Cox proportional hazards regression. Based on the results of multivariate analyses, a nomogram was built to predict the overall survival (OS) of patients with pelvic ES. The performance of the nomogram was evaluated by the concordance index (C-index).

**Results:**

A total of 267 cases diagnosed between 2004 and 2016 were included in the study. Univariate and multivariate analyses showed that patients who were younger, white, had a localized tumor stage, or underwent surgery were associated with improved prognoses, while no significant differences were observed in OS based on sex, tumor size, or radiotherapy. A nomogram was developed and the C-index was 0.728, indicating adequate performance for survival prediction.

**Conclusions:**

Age, race, tumor stage, and surgery were identified as independent prognostic factors for the OS of pelvic ES. The nomogram developed in this study can individually predict 3- and 5-year OS in patients with pelvic ES.

## Introduction

Ewing sarcoma (ES) is a rare malignancy that accounts for ~8% of all primary malignant bone tumors with a peak incidence in children and adolescents (Ranft et al., 2017). In the USA, the annual incidence of ES has remained at about 0.1/100,000 for the past 30 years (Wan, Huang & Chen, 2018). Additionally, ~25% of cases originate in the pelvis, which is considered to be the second most common site involved in ES (Zhu et al., 2018). Due to the development of local treatments and systemic chemotherapies, the survival of ES has been greatly improved in recent years (Bosma et al., 2018). However, despite advances in treatment, the prognosis of pelvic ES remains poor compared to ES at other primary sites (Mounessi et al., 2013). In the pelvis, there is a lack of anatomic barriers of tumor diffusion, and the pelvis is also adjacent to the internal organs and neurovascular bundles, which makes local control difficult (Fan et al., 2017). Notably, the local recurrence rate of patients with pelvic ES following treatment is 20–30%, which is significantly higher than that of extremities (13%) (Albergo et al., 2018; Raciborska et al., 2014). Moreover, due to the rarity of pelvic ES, there is a lack of analyses of prognostic factors, and determinations of the optimal local treatments are challenging (Ahmed et al., 2017). Therefore, we sought to develop a prognostic model incorporating all prognostic factors to individually predict survival of patients with pelvic ES based on large samples.

Nomograms are graphic depictions of predictive statistical models that have been used widely to accurately predict the survival of cancer patients, including those with lung cancer, chondrosarcoma, and breast cancer, among others (Dihge, Bendahl & Ryden, 2017; Song et al., 2018; Young et al., 2017). Nevertheless, to our knowledge, the application of nomograms in patients with pelvic ES has not been performed. The Surveillance, Epidemiology, and End Results (SEER) program published clinical information of ES patients that allows for the prognosis of pelvic ES (Zeng et al., 2015). In this study, we took advantage of those data to identify risk factors affecting the overall survival (OS) of patients with pelvic ES, and a nomogram was developed to visually predict the prognosis of this disease.

## Patients and methods

### Ethics statement

This study was based on the data released from an online publicly available SEER database, and data extracted from SEER were identified as nonhuman study. Thus, it was deemed exempt by the Ethics Committee of West China Hospital, Sichuan University (Chengdu, China).

### Data selection

**Figure 1:**
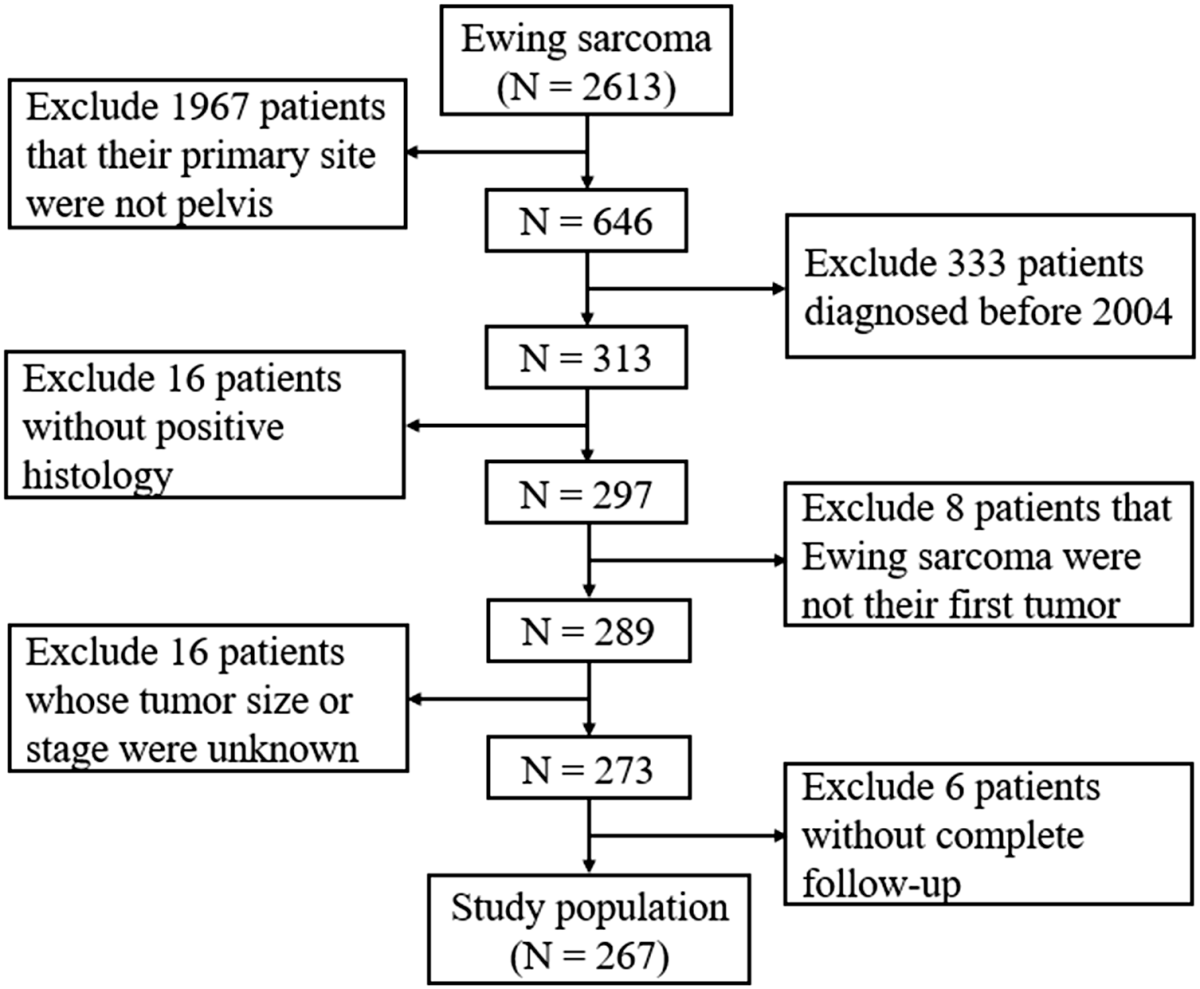
Flow diagram of selecting process in the SEER database.

SEER*Stat software version 8.3.5 (NCI, Bethesda, MD, USA) was used to obtain data from the SEER database of pelvic ES patients who were diagnosed and treated between 2004 and 2016, as patients diagnosed before 2004 had insufficient information on tumor size in this database. The database is population-based and contains data from 18 states in the USA, with annual updates on the clinical information of cancer patients, including age at diagnosis, sex, race, tumor characteristics, treatment, follow-up, and survival (Doll, Rademaker & Sosa, 2018). As shown in Fig. 1, the selection process was conducted to obtain sufficient eligible data from the database. Briefly, patients who had the International Classification of Diseases for Oncology, third edition histology/behavior code 9260/3-Ewing sarcoma with the primary site C41.4-Pelvic bones were included. Patients were excluded based on the following: (1) those without positive histological confirmations, (2) the pelvic ES was not their first tumor; (3) the tumor size or tumor stage in the database was unknown, or (4) the complete dates of follow-up were unavailable or survival did not exceed 0 days.

### Variables

Patients’ clinical characteristics, including age at diagnosis, sex, race, tumor size, stage, treatment, vital status, and survival months were collected. A subset of characteristics was categorized for further analysis. The age at diagnosis was categorized as ≤9, 10–17, and ≥18 years (Ogura et al., 2015). Race was categorized as white or others (Black, American Indian/Alaskan Native, Asian/Pacific Islander). Tumor sizes were grouped based on the largest tumor diameter (≤ or >8 cm) from the variable “CS tumor size (2004+)” (Davenport et al., 2016). The tumor stage was divided into three groups based on the variable “SEER historical stage A,” including localized, regional, and distant. According to the 2018 version of the Summary Stage Manual provided by SEER (https://seer.cancer.gov/tools/ssm/), localized indicated that the tumor was confined to the pelvis or one to two pelvic segments were involved without known extraosseous extensions. Regional indicated one to two pelvic segments were involved with extraosseous extensions, and without distant metastasis.

### Statistical analysis

For each patient variable, the prognostic effect was clarified using SPSS 24.0 (IBM Corp., Armonk, NY, USA). The Kaplan–Meier method was performed to construct cumulative survival curves and was compared using the log-rank test. OS was chosen as the primary survival outcome in this study. OS was defined as the period from diagnosis to death from any causes. Cox proportional hazards regression was used to identify significant prognostic factors, and variables with *P*-values < 0.05 in univariate analyses were further analyzed in multivariate analyses (Hu et al., 2019; Pan et al., 2019; Wang et al., 2019). Following this, a prognostic nomogram based on the results of the multivariate analyses was constructed using the rms package in R software, version 3.5.1 (R Core Team, 2018). The maximum score for each prognostic factor was set at 10. The performance of the nomogram was evaluated by the concordance index (C-index) ranging from 0.5 (a very poor model) to 1.0 (a perfect model). Generally, a C-index >0.7 indicates a good model (Liang et al., 2015). The calibration curve was based on 1,000 bootstrap replicates and was derived to compare the nomogram-predicted survival to the actual survival. A two-tailed *P* < 0.05 was defined as a significant difference.

## Results

### Baseline characteristics of patients

The SEER database contained 2,613 patients with ES, of which 24.7% (646/2,613) were pelvic ES. Following assessments of the aforementioned inclusion and exclusion criteria, a total of 267 patients were included in the study (Fig. 1). The 267 pelvic ES patients were located in California (112, 41.9%), Connecticut (15, 5.6%), Georgia (26, 9.7%), Hawaii (3, 1.1%), Iowa (8, 3.0%), Kentucky (12, 4.5%), Louisiana (10, 3.8%), Michigan (9, 3.4%), New Jersey (32, 12.0%), New Mexico (6, 2.2%), Utah (13, 4.9%), and Washington (21, 7.9%).

The 267 patients had a median age of 16 years and an interquartile range of 12–22 years. As shown in Table 1, the sample set consisted of 174 (65.2%) male and 93 (34.8%) female patients. In the sample population, whites accounted for the majority. Among the patients, 97 (36.3%) had a tumor size of no more than eight cm, while 170 (63.7%) had a tumor size exceeding eight cm. A total of 39 patients were at the localized stage (14.6%), while 102 were at the regional stage (38.2%), and 126 were at the distant stage (47.2%). Additionally, surgery was performed in 80 (30.0%) patients and radiotherapy was performed in 55 (20.6%) patients.

**Table 1:** Patient characteristics and 3- and 5-year overall survival rates.

Characteristics	Number of patients	Percent	3-year OS (%)	5-year OS (%)
Age (years)				
≤9	32	12.0	76.7 ± 9.3	76.7 ± 9.3
10–17	118	44.2	69.4 ± 4.5	56.7 ± 5.1
≥18	117	43.8	48.3 ± 5.1	37.1 ± 5.2
Sex				
Male	174	65.2	60.4 ± 4.1	49.2 ± 4.3
Female	93	34.8	62.5 ± 5.5	52.6 ± 5.9
Race				
White	232	86.9	63.1 ± 3.4	51.6 ± 3.7
Others	35	13.1	46.4 ± 9.9	41.2 ± 10.0
Tumor size (cm)				
≤8	97	36.3	65.3 ± 5.3	54.9 ± 5.7
>8	170	63.7	58.7 ± 4.2	47.6 ± 4.4
Tumor stage				
Localized	39	14.6	89.9 ± 5.6	77.6 ± 8.1
Regional	102	38.2	69.6 ± 5.0	60.2 ± 5.5
Distant	126	47.2	45.7 ± 4.9	33.2 ± 5.1
Surgery				
No	187	70.0	55.8 ± 4.0	42.5 ± 4.3
Yes	80	30.0	73.4 ± 5.4	66.7 ± 5.9
Radiotherapy				
No	212	79.4	60.2 ± 3.7	47.8 ± 4.1
Yes	55	20.6	64.8 ± 6.7	58.2 ± 7.0
Surgery with radiotherapy				
No surgery	8	3.0	50.0 ± 17.7	18.8 ± 15.8
Underwent surgery	47	17.6	67.5 ± 7.2	64.9 ± 7.3
Surgery without radiotherapy				
No surgery	179	67.0	56.1 ± 4.1	44.0 ± 4.4
Underwent surgery	33	12.4	84.0 ± 7.4	69.9 ± 9.7

### Survival analysis

**Figure 2:**
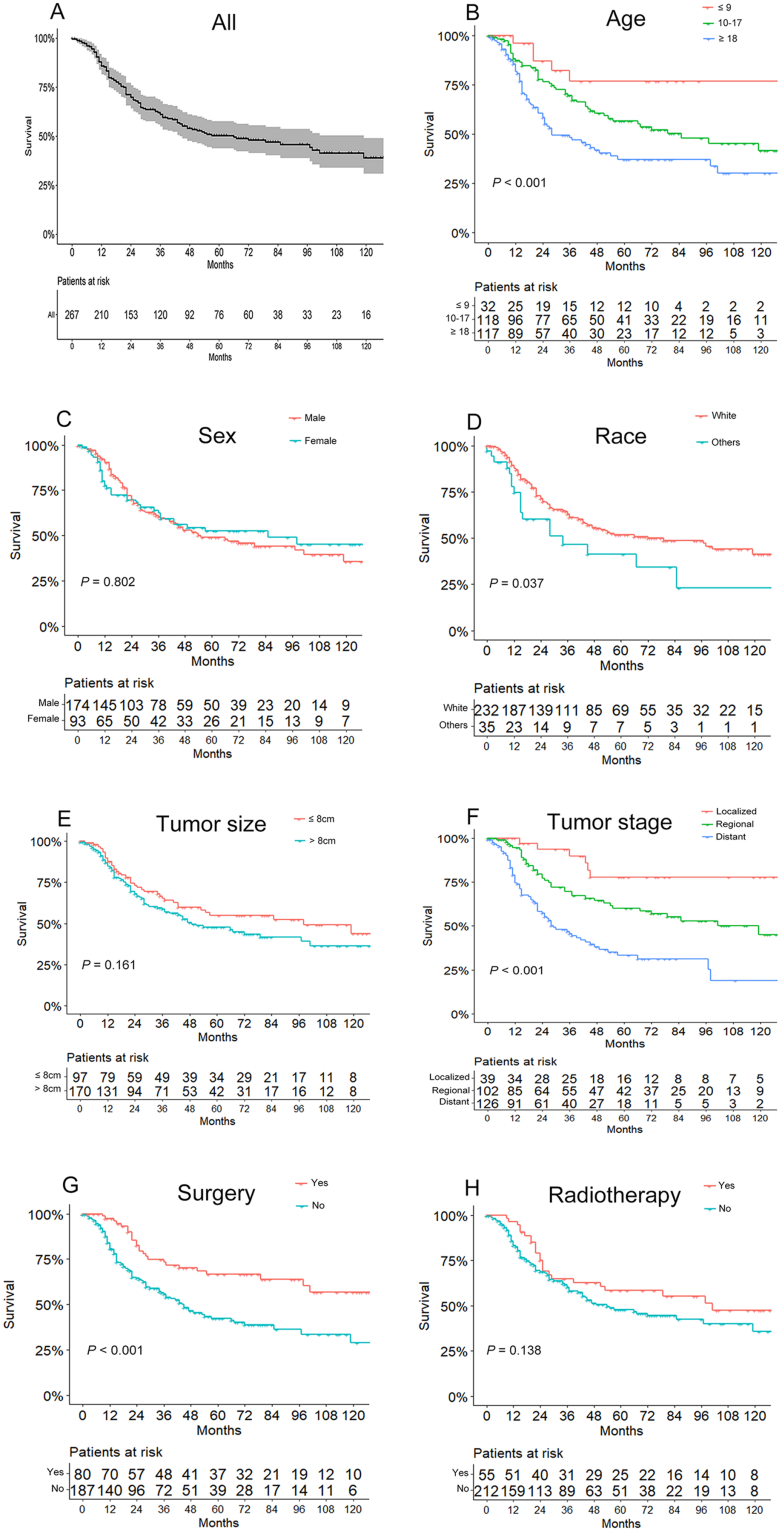
Kaplan–Meier curves of overall survival for patients based on (A) all included patients, (B) age, (C) sex, (D) race, (E) tumor size, (F) tumor stage, (G) use of surgery, and (H) use of radiotherapy.

The OS of the 267 patients ranged from 1 to 142 months, with a median of 28 months. As shown in Fig. 2A, the overall 3- and 5-year survival rates were 61.1% and 50.3%, respectively. And the 3- and 5-year survival rates for different subgroups are shown in Table 1. Based on Kaplan–Meier curves and log-rank analyses, younger patients (*P* < 0.001, Fig. 2B), whites (*P* = 0.037, Fig. 2D), patients with localized tumor stages (*P* < 0.001, Fig. 2F), and those who underwent surgery (*P* < 0.001, Fig. 2G) were associated with better prognoses, while no significant differences were observed in OS when considering sex (*P* = 0.802, Fig. 2C), tumor size (*P* = 0.161, Fig. 2E), and radiotherapy treatment (*P* = 0.138, Fig. 2H). Besides, surgery showed an association with better prognosis in patients without radiotherapy, but had an unfavorable influence for radiotherapy patients. The details of the correlation between survival and factors mentioned above are shown in Table 1.

To control for confounding variables, the risk factors identified by univariate analyses were further explored in multivariate analyses. As shown in Table 2, Cox proportional hazards regression analyses revealed that age (*P* = 0.006), race (*P* = 0.018), tumor stage (*P* < 0.001), and surgery (*P* = 0.030) were the independent risk factors for prognosis.

**Table 2:** Univariate and multivariate Cox proportional hazards regression analysis.

Characteristics	Univariate analysis			Multivariable analysis		
	HR	95% CI	*P*-value	HR	95% CI	*P*-value
Age (years)			0.001			0.006
≤9	Reference			Reference		
10–17	2.426	[0.967–6.086]		2.110	[0.838–5.315]	
≥18	4.097	[1.646–10.197]		3.366	[1.340–8.457]	
Sex			0.804	Not included		
Male	Reference					
Female	0.952	[0.646–1.403]				
Race			0.040			0.018
White	Reference			Reference		
Others	1.693	[1.023–2.802]		1.867	[1.112–3.135]	
Tumor size (cm)			0.165	Not included		
≤8	Reference					
>8	1.314	[0.894–1.932]				
Tumor stage			<0.001			<0.001
Localized	Reference			Reference		
Regional	2.665	[1.128–6.297]		2.376	[1.002–5.633]	
Distant	5.933	[2.570–13.694]		5.048	[2.181–11.686]	
Surgery			<0.001			0.030
No	Reference			Reference		
Yes	0.447	[0.286–0.698]		0.603	[0.381–0.953]	
Radiotherapy			0.143	Not included		
No	Reference					
Yes	0.714	[0.454–1.121]				

### Predictive nomogram

**Figure 3:**
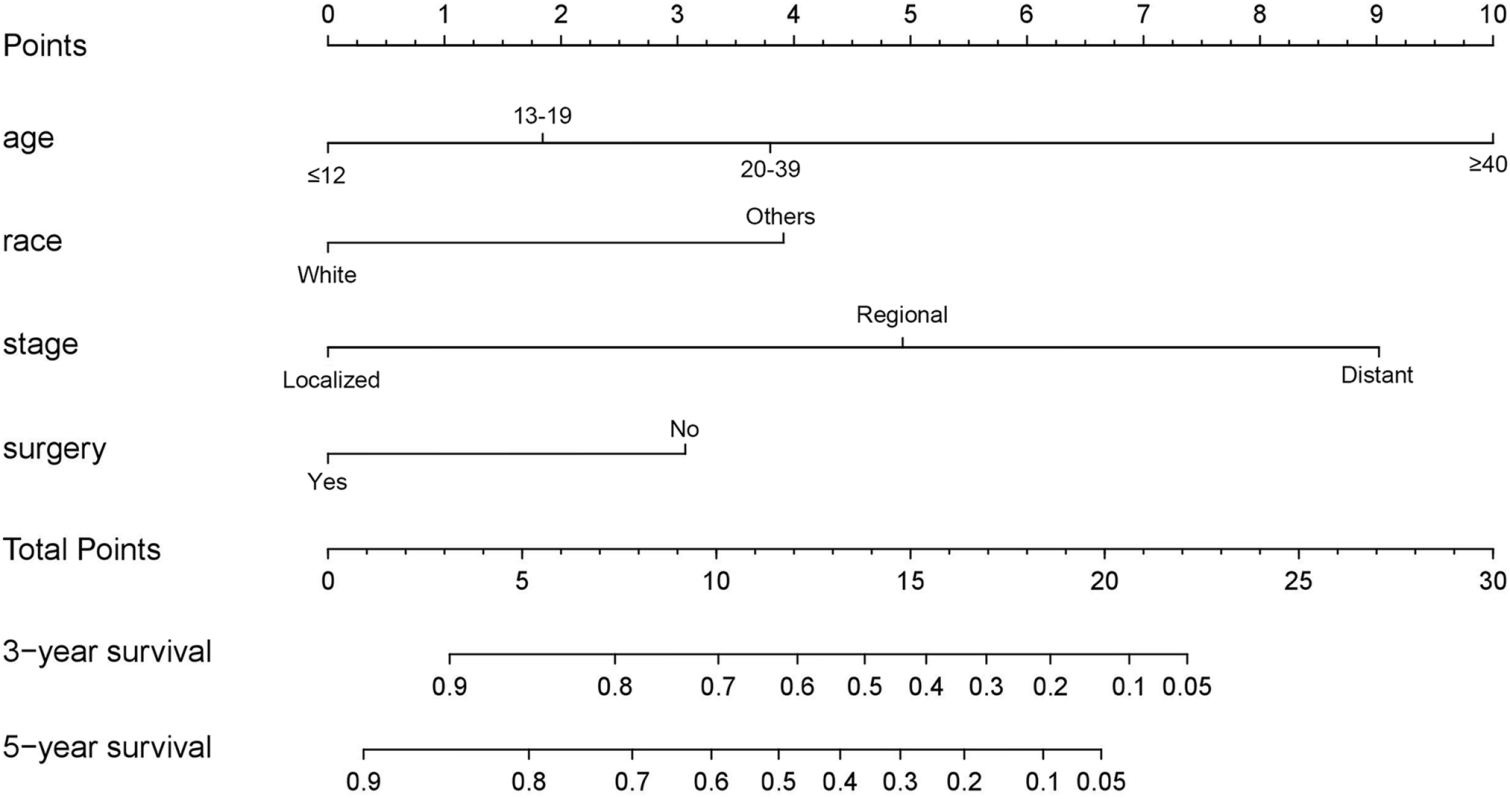
Nomogram to predict 3- and 5-year overall survivals in patients with pelvic Ewing sarcoma.

**Figure 4:**
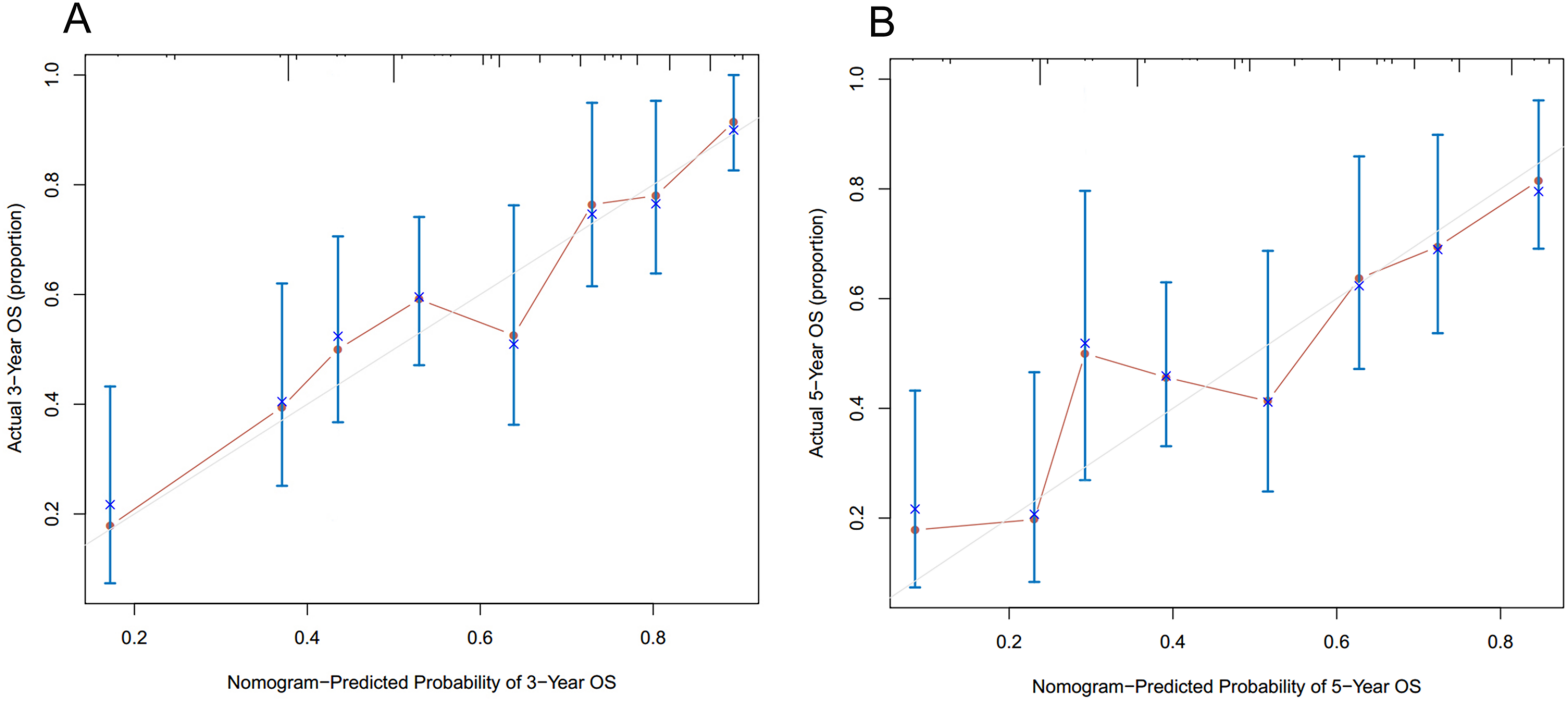
Calibration curves of the nomogram predicting (A) 3-year and (B) 5-year overall survivals in patients with pelvic Ewing sarcoma.

As shown in Fig. 3, the nomogram for predicting 3- and 5-year OS rates of pelvic ES was constructed based on the significant risk factors identified by multivariate analyses. To calculate the 3- and 5-year OS rates, each factor was first evaluated using the points scale at the top of the nomogram, which were then summed for each factor. Following this, the point scale located at the base of the nomogram were used to determine the 3- and 5-year OS rates. Through bootstrap resampling validations, calibration plots were determined (illustrated in Fig. 4) showing excellent agreement with actual survival. The C-index of the nomogram was 0.728, suggesting good prediction accuracies for OS in patients with pelvic ES.

## Discussion

Personalized medicine is playing an increasingly important role in cancer therapies (Thewes et al., 2017). By establishing prognostic models, treatment stratifications can be improved to avoid over- or under-treatment, while risk- and response-adaptive treatment strategies can be made earlier (Bosma et al., 2018). Nomograms are widely-accepted prognostic models that have the ability to incorporate various prognostic factors for predicting individual survivals (Iasonos et al., 2008). Although pelvic ES is a highly malignant bone tumor with poor prognostic options, for which no nomogram has been developed. This study used data from the SEER database to identify several independent prognostic factors of pelvic ES using univariate and multivariate analyses. A nomogram with good prediction performance for 3- and 5-year OS rates was developed based on four significant factors including age, race, tumor stage, and surgery.

Age is generally considered to be correlated with the prognosis of multiple malignant diseases (Adam et al., 2016; Wensink, 2016). Consistent with that hypothesis, the analysis of demographic characteristics in this study also identified age as an important prognostic factor for pelvic ES. The median age at diagnosis of patients with pelvic ES was 16 years, and as was consistent with previous studies (Lin et al., 2019; Ng et al., 2015), younger patients had more favorable survivals. Compared to patients of other races, white patients had a better survival (Davenport et al., 2016; Worch et al., 2010). A possible explanation for this was that treatment disparities and/or delays in diagnosis may have had an impact on different outcomes (Fiscella & Sanders, 2016); also, the biological behavior of tumors may also differ between races (Kadan-Lottick et al., 2003).

Survival rates based on tumor size have been inconsistent in previous studies. The majority of such studies supported that larger tumors were not conducive to the survival of ES patients (Hesla et al., 2016; Raciborska et al., 2014; Wan, Huang & Chen, 2018). In contrast, our research showed that tumor size did not affect the survival of pelvic ES patients, which was consistent with multiple other studies (Donati et al., 2007; Yock et al., 2006). One possible explanation for those findings is that tumor size during diagnosis is associated with the treatment type administered, which can affect survival. For example, combined-modality therapy for local control was more likely to be chosen by patients and clinicians, and thus, the deleterious effects of large tumors was partially reduced (Yock et al., 2006). Tumor stage was also an independent prognostic factor for pelvic ES in this study, and the presence of distant stages at diagnosis could result in poorer survival rates than ES at localized or regional stages. Such a trend further demonstrates the importance of improving early diagnoses, since ES originating in the pelvis is prone to delayed detection due to vague early symptoms (Kadhim et al., 2018).

Regardless of the challenges associated with pelvic resections, recurrence, or the loss of physical functions, there have been recent trends favoring surgery to treat pelvic ES (Puri et al., 2012). Our research also revealed that surgical treatments were associated with better outcomes for pelvic ES. When considering tumor size, stage, and anatomical location, the decision of surgical resection should be individualized to achieve a wide resection and negative margin (Hosalkar & Dormans, 2007). Several previous studies compared radiotherapy to surgery, and found that surgery was superior for event-free survival, OS, and local control (Ahmed et al., 2017; Choi et al., 2015; DuBois et al., 2015). Collaboration between surgeons and oncologists is important to determine the pros and cons of each patient’s postoperative reconstruction so that pelvic ES patients undergoing surgery can experience better prognoses with good physical functions (Kadhim, Womer & Dormans, 2017).

There are some potential limitations to this study. First, this study is retrospective and may require large randomized controlled trials to further validate the findings. Second, the data for several variables, including surgical margins, local recurrence, and chemotherapy were insufficient in the SEER database, and were not evaluated. Finally, the C-index is a good nomogram validation tool, however, the findings would be more reliable if external validations were performed using other independent large-scale datasets. Despite such limitations, we constructed an effective and accurate prognostic nomogram, which individually predicted the survival of patients with pelvic ES.

## Conclusions

We identified age, race, tumor stage, and surgery as independent prognostic factors for pelvic ES, while sex, tumor size and radiotherapy were not significant risk factors. A nomogram that integrated all such independent prognostic factors was built and had good prediction accuracies. The developed nomogram can provide clinicians with access to individual assessments of 3- and 5-year OS in pelvic ES patients.

## Supplemental Information

10.7717/peerj.7710/supp-1 Supplemental Information 1 Clinipathological characteristics of pelvic ES.

## Additional Information and Declarations

### Data Availability

The following information was supplied regarding data availability: The raw measurements are available in the Supplemental File.
